# Association of VDR BsmI polymorphism and vitamin D status with osteoarthritis susceptibility

**DOI:** 10.1186/s12920-026-02339-0

**Published:** 2026-03-02

**Authors:** Shawnim M. Maaruf, Dara K. Mohammad, Treska S. Hassan, Azhin D. Aziz, Raya Kh. Yashooa, Haween T. Hassan, Sarkawt Sarteeb Fattah Agha, Rebar Nadhem A. Daham, Mukhlis H. Aali, Suhad A. Mustafa

**Affiliations:** 1 General Directorate of Scientific Research Center, Salahaddin University-Erbil, KRG-Iraq, Erbil, 44001 Iraq; 2 Center for Hematology and Regenerative Medicine (HERM), Department of Medicine Huddinge, Karolinska Institutet, Stockholm, SE-141 83 Sweden; 3 College of Agricultural Engineering Sciences, Salahaddin University-Erbil, Erbil, KRG 44002 Iraq; 4 Directorate of Health-Erbil, Rizgary Teaching Hospital, Erbil, KRG 44001 Iraq; 5 Department of Biology, College of Education for Pure Sciences, University of Al- Hamdaniya, Mosul, 41002 Iraq; 6 College of Dentistry, Hawler Medical University, Erbil, KRG 44002 Iraq; 7 Department of Microbiology, Shar Hospital, Erbil, Iraq; 8 Kurdistan Higher Council for medical specialties- Erbil, KRG-Iraq, Erbil, 44001 Iraq; 9 Department of Biology, College of Science, Salahaddin University-Erbil, KRG-Iraq, Erbil, 44001 Iraq

**Keywords:** Vitamin D receptor, VDR polymorphism, Osteoarthritis, Bioinformatics, Kurdish population

## Abstract

**Background:**

Osteoarthritis (OA) is a chronic degenerative joint disease influenced by genetic, environmental, and immunological factors. Vitamin D exerts immunomodulatory and anti-inflammatory effects through the vitamin D receptor (VDR), and genetic variation in the VDR gene may influence susceptibility to OA. However, data from Middle Eastern and Kurdish populations remain limited.

**Objective:**

This study aimed to evaluate the association between serum vitamin D status and four common VDR gene polymorphisms (FokI, ApaI, TaqI, and BsmI) in Kurdish adults with knee osteoarthritis.

**Methods:**

A hospital-based case–control study was conducted, including 100 OA patients and 100 healthy controls recruited in Erbil, Iraq. Serum vitamin D levels were measured biochemically, and VDR polymorphisms were genotyped using PCR-RFLP and sequencing. Association analyses were performed for polymorphic loci using univariate logistic regression.

**Results:**

No allelic or genotypic variation was detected at the FokI (rs2228570), ApaI (rs7975232), or TaqI (rs731236) loci, indicating allele fixation in this population. In contrast, the BsmI (rs1544410) polymorphism exhibited significant variability. The AA genotype was significantly more frequent among OA patients than controls and was associated with increased odds of OA (OR = 2.26, 95% CI = 1.21–4.23; *p* = 0.006).

**Conclusions:**

The findings indicate that the VDR BsmI polymorphism is associated with knee osteoarthritis in the Kurdish population, whereas FokI, ApaI, and TaqI loci were non-polymorphic. These results highlight population-specific genetic variation within the VDR gene and underscore the need for larger studies incorporating functional validation to clarify the biological relevance of BsmI variation in osteoarthritis.

## Introduction

 Osteoarthritis (OA) is the most common degenerative joint disease and a leading cause of chronic pain and disability worldwide, affecting populations in both developed and developing countries. It is characterized by progressive loss of articular cartilage, subchondral bone sclerosis, osteophyte formation, synovial inflammation, and deterioration of joint function, ultimately resulting in reduced mobility and quality of life [1]. The global burden of OA is substantial, affecting an estimated 300 million people worldwide, with symptomatic knee OA reported in approximately 10% of men and 13% of women aged 60 years and older [2, 3]. According to the Global Burden of Disease Study, OA ranks among the leading causes of Years Lived with Disability (YLD), reflecting its considerable social and economic impact [3]. The pathophysiology of OA is multifactorial and involves complex interactions between biomechanical stress, chronic low-grade inflammation, and metabolic dysregulation. Established risk factors include age, obesity, biomechanical overload, genetic variability, and sex, with post-menopausal women exhibiting increased susceptibility [4, 5]. Disease progression is driven by sustained inflammatory signaling, chondrocyte apoptosis, and progressive degradation of the extracellular matrix (ECM), processes that collectively contribute to cartilage destruction and joint dysfunction. Recent studies have highlighted the role of inflammatory mediators in promoting apoptotic pathways in chondrocytes and accelerating ECM breakdown, thereby amplifying OA progression [6]. At the molecular level, synovial inflammation and immune cell infiltration, including macrophages and T cells, promote the secretion of pro-inflammatory cytokines such as IL-1β, TNF-α, and IL-6. These cytokines activate chondrocytes to produce matrix metalloproteinases (MMPs), leading to cartilage degradation and further joint damage [7–9]. Altered bone remodeling mediated by osteoblast and osteoclast dysfunction also contributes to subchondral bone sclerosis and pain severity. Vitamin D is a fat-soluble secosteroid synthesized in the skin from 7-dehydrocholesterol following exposure to ultraviolet B (UVB) radiation, forming cholecalciferol (vitamin D₃), while ergocalciferol (vitamin D₂) is obtained from dietary sources. Both forms undergo hepatic hydroxylation by CYP2R1 and CYP27A1 to produce 25-hydroxyvitamin D [25(OH)D], the primary circulating biomarker of vitamin D status [10, 11]. Subsequent renal hydroxylation yields the biologically active metabolite 1,25-dihydroxyvitamin D [1,25(OH)₂D], which exerts its effects through binding to the vitamin D receptor (VDR) [11–14]. Emerging evidence suggests that genetic variation in the VDR gene may influence susceptibility to OA [15]. The VDR gene is located on chromosome 12q13.1 and contains several commonly studied polymorphisms, including (FokI rs2228570 T > C, BsmI rs1544410 G > A, ApaI rs7975232 G > T, and TaqI rs731236 T > C). Among these, the FokI polymorphism has been reported to alter the translation initiation site, resulting in VDR protein isoforms with differing transcriptional activity, which may influence downstream immunomodulatory effects [16, 17]. While some studies have reported associations between VDR polymorphisms and OA risk, findings remain inconsistent across populations [15, 18], underscoring the need for population-specific investigations. Despite growing interest in vitamin D and VDR polymorphisms in OA, data from Middle Eastern and Kurdish populations remain limited. Therefore, we conducted a case–control study to evaluate the association between serum vitamin D status and genetic variation in the *VDR* gene, including (FokI, BsmI, ApaI, and TaqI) polymorphisms, in Kurdish adults aged over 40 years with knee osteoarthritis. This study aims to address gaps in population-specific genetic data and contribute to a more nuanced understanding of the potential role of VDR gene variation in OA within this understudied population.

## Materials and methods

### Ethical statement

The study was approved by the Human Ethical Committee (HEC) at Salahaddin University-Erbil (KRG-Iraq), (Registration no: 4 S/275, 22/2/2021). Informed consent was taken from the participants before joining the study. The experimental research was conducted incompliance with institutional human ethical committee norms.

### Design and participants

This hospital-based, age-comparable case–control study included 100 patients with clinically diagnosed osteoarthritis (OA) and 100 healthy controls recruited from caregivers attending orthopedic clinics and semi-government-funded hospitals in Erbil, Kurdistan Region, Iraq. The diagnosis of OA was based on clinical evaluation supported by radiographic findings, and inclusion criteria comprised age ≥ 40 years in either sex and chronic knee pain lasting more than six months. Control participants were individuals without a history of osteoarthritis, chronic joint pain, or inflammatory arthritis, confirmed by clinical assessment. Participants with autoimmune diseases, including systemic lupus erythematosus, antiphospholipid syndrome, or rheumatoid arthritis, as well as those with a history of fracture or joint surgery within six months prior to enrollment, were excluded from both groups.

### Sample collection and processing

Peripheral blood was collected from the participants (5 mL) in EDTA-coated lavender tubes after a 15-minute rest. Once taken, blood samples were stored at -20 °C for further use in molecular techniques, including genomic DNA extraction and PCR analysis.

### Human genomic DNA extraction

Human Genomic DNA was extracted from lymphocytes using the Jena Bioscience spin-column kit (Germany) following the manufacturer’s protocol. The quantity and integrity of the extracted genomic DNA were assessed using a NanoDrop™ spectrophotometer (Thermo Scientific, USA), which exhibited DNA purity values ranging from (1.7 to 2.0) and concentrations between (60-320ng/µL). Genomic DNA integrity was confirmed by 1.5% agarose gel electrophoresis [18]. The genetic analysis was conducted at the Salahaddin University Research Center (SURC), and the Nobel Medical Genetics Laboratory in Erbil City.

### Genotyping of VDR polymorphism

Genomic DNA was amplified to investigate four polymorphic sites (SNPs) on the *VDR* gene using the forward and reverse primers described and detailed in Table 1 [19]. The selected polymorphisms (FokI, BsmI, ApaI, and TaqI) were analyzed, and their genotypes were determined and assessed using polymerase chain reaction (PCR) techniques. The *VDR* alleles FokI, BsmI, ApaI and TaqI are located on exon 2, intron 8, and exon 9 in the *VDR* gene, respectively. Retrieve the *VDR* gene sequence from the gene library, use Primer3Plus to design four *VDR* allele polymorphic (SNPs) site primers based on the sequences, and use NCBI’s Blast and Unipro UGENE software to test and check the specificity of the primers. These primers were purchased from Macrogen Inc. (Seoul, South Korea). The primers were provided in lyophilized form and dissolved in deionized distilled water to obtain 100 µM stock concentrations from which working primer solutions were prepared by adding (10–90 µL) of deionized distilled water.

The PCR mixture 25 µl, consisting of 1 µl of each primer (forward and reverse 10 µM), 12.5 µl of *Ampliqon* Red Dye Master Mix 2X(Ampliqon/ Denmark) (containing *Taq DNA polymerase* and the NH^4+^ buffer system, dNTPs and magnesium chloride MgCl_2_) and genomic DNA 3 µl (30 ng/ µl) and finally completed to 25 µl by adding 7.5 µl of deionized distilled water. The PCR amplification conditions and RFLP-PCR method analyses for the four polymorphic sites (SNPs) of the *VDR gene* were adapted from the published protocol by Rasoul et al. [19], with slight modifications [19]. Separation of the PCR products was performed using horizontal gel electrophoresis on a 2% agarose gel prepared by adding 2gm of agarose and mixing it with 100 ml of 1X TBE buffer. The gel was visualized using a UV-transilluminator after staining with the DNA dye Safe-stain. UV Transillumination (UVP, USA) at (λ = 240–366 nm) wavelength was used to visualize the DNA banding pattern [20]. The gel was illuminated from below by placing it on the transilluminator glass. The 100 bp DNA Ladder (GeneDireX, Inc., Taiwan) was used as a molecular marker, and photographs were captured with a digital photography camera (Canon G12) [21].

**Table 1:** Primer Sets for VDR Gene Polymorphic Sites (FokI, ApaI, TaqI, BsmI) and PCR Amplicon Sizes [19]

VDR Gene Sites	Primer Sequences 5’-3’	PCR Products
*FokI* (C > T, Rs10735810)	Forward AGCTGGCCCTGGCACTGACTATGCTCT Reverse ATGGAAACACCTTGCTTCTTCTCCCT	265 bp
*ApaI* (G > T, Rs7975232)	Forward CAGAGCATGGACAGGGAGCAAG Reverse GCA ACTCCTCATGGCTGAGGTCTCA	740 bp
*TaqI* (T > C, Rs731236)		
*BsmI* (A > G, rs1544410)	Forward AACCAGCGGGAAGAGGTCAAGGG Reverse CAACCAAGACTACAAGTACCGCGTCAGTGA	870 bp

For Sanger sequencing, sample preparation involved using approximately 30–35 µL of each PCR product for *VDR allele* polymorphic sites and 25–30 µL of reverse primers for each specific region (exon 2, intron 8, and exon 9). These samples were then sent to Macrogen Inc. in Seoul, South Korea. Sequencing was performed using the Big Dye Terminator method on an automated 3130 Genetic Analyzer (Applied Biosystems; Thermo Fisher Scientific, Inc.).

### Statistical analysis

All statistical analyses were performed using SPSS version 28. Genotype distributions in the control group were assessed for Hardy–Weinberg equilibrium (HWE) using Pearson’s chi-square (χ²) test, with a *p-*value > 0.05 indicating compliance with HWE expectations. Genotype and allele frequency distributions between (OA) cases and controls were compared using two-sided chi-square tests or Fisher’s exact tests. For polymorphic loci, associations with OA were evaluated using univariate logistic regression to estimate crude odds ratios (ORs) and 95% confidence intervals (CIs). Genetic models were explored descriptively where genotype variation was present. A *p*-value < 0.05 was considered statistically significant.

### Bioinformatics and sequence conservation analysis

Sequence conservation analysis of the vitamin D receptor (VDR) gene was performed using publicly available nucleotide and protein reference sequences obtained from the NCBI database. Multiple sequence alignments were conducted using standard alignment tools to assess evolutionary conservation across orthologous VDR sequences. No structural modeling, molecular dynamics simulations, or mRNA stability prediction algorithms were applied. Therefore, any discussion of potential functional implications is speculative and intended solely to provide biological context.

## Results

A total of 200 subjects were included in the study, comprising 100 osteoarthritis (OA) cases and 100 healthy controls. Table 2 summarizes the demographic and clinical characteristics of the participants. The mean age of OA cases was slightly lower than that of controls (49.08 ± 9.00 vs. 52.44 ± 8.74 years), with a marginally non-significant difference (*p* = 0.06). A statistically significant difference in sex distribution was observed, with a higher proportion of males in the OA group compared with controls (58/42 vs. 36/64; *p* = 0.0029). Body mass index was comparable between groups (25.37 ± 2.21 vs. 26.52 ± 2.90 kg/m²; *p* = 0.21). Serum vitamin D levels were significantly higher in OA cases than in controls (20.88 ± 8.06 vs. 18.75 ± 9.00 ng/mL; *p* = 0.0094). No significant differences were observed for calcium levels or history of previous bone fracture between the two groups.

**Table 2:** Anthropometric and Lifestyle Characteristics of Study Participants

Parameter	OA Cases (*n* = 100)	Controls (*n* = 100)	*P*-value
Age (years, Mean ± SD)	49.08 ± 9.00	52.44 ± 8.74	0.06
Sex (Male/Female)	58 / 42	36 / 64	0.0029
BMI (kg/m², Mean ± SD)	25.37 ± 2.21	26.52 ± 2.90	0.21
Vitamin D Level (ng/mL, Mean ± SD)	20.88 ± 8.06	18.75 ± 9.00	0.0094
Calcium Level (mg/dL, Mean ± SD)	8.84 ± 0.42	8.92 ± 0.45	0.40
Previous Bone Fracture (Yes/No)	39 / 61	31 / 69	0.29

BMI = body mass index. *p*-values < 0.05 were considered significant

### *FokI* polymorphism (rs2228570)

**Fig. 1:**
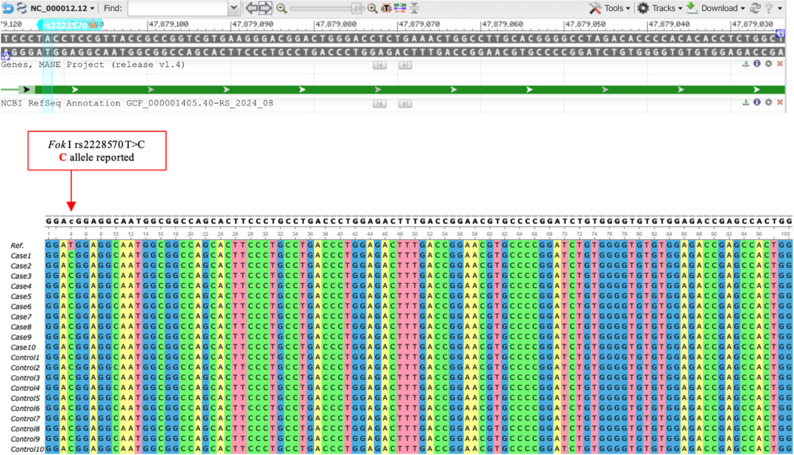
Sequence alignment of VDR *FokI* (rs2228570) polymorphism. Sequence alignment generated using Unipro UGENE v51.0 shows the T > C allele substitution; all study participants carried the C allele, indicating the absence of polymorphism at this site. The reference sequence from a genomic database (NCBI) is displayed at the top, with the annotated FokI polymorphism (rs2228570) indicating a T > C nucleotide change at chromosome 12: 47,879,112 (GRCh37)

Sequence analysis demonstrated that all study participants, including both osteoarthritis cases and controls, carried the C allele at the FokI locus (Fig. 1). No allelic or genotypic variation was detected, indicating that the FokI polymorphism was non-polymorphic (fixed) in this study population. Consequently, no association analysis was performed for this locus.

### *Bsm I* polymorphism (rs1544410)

**Fig. 2:**
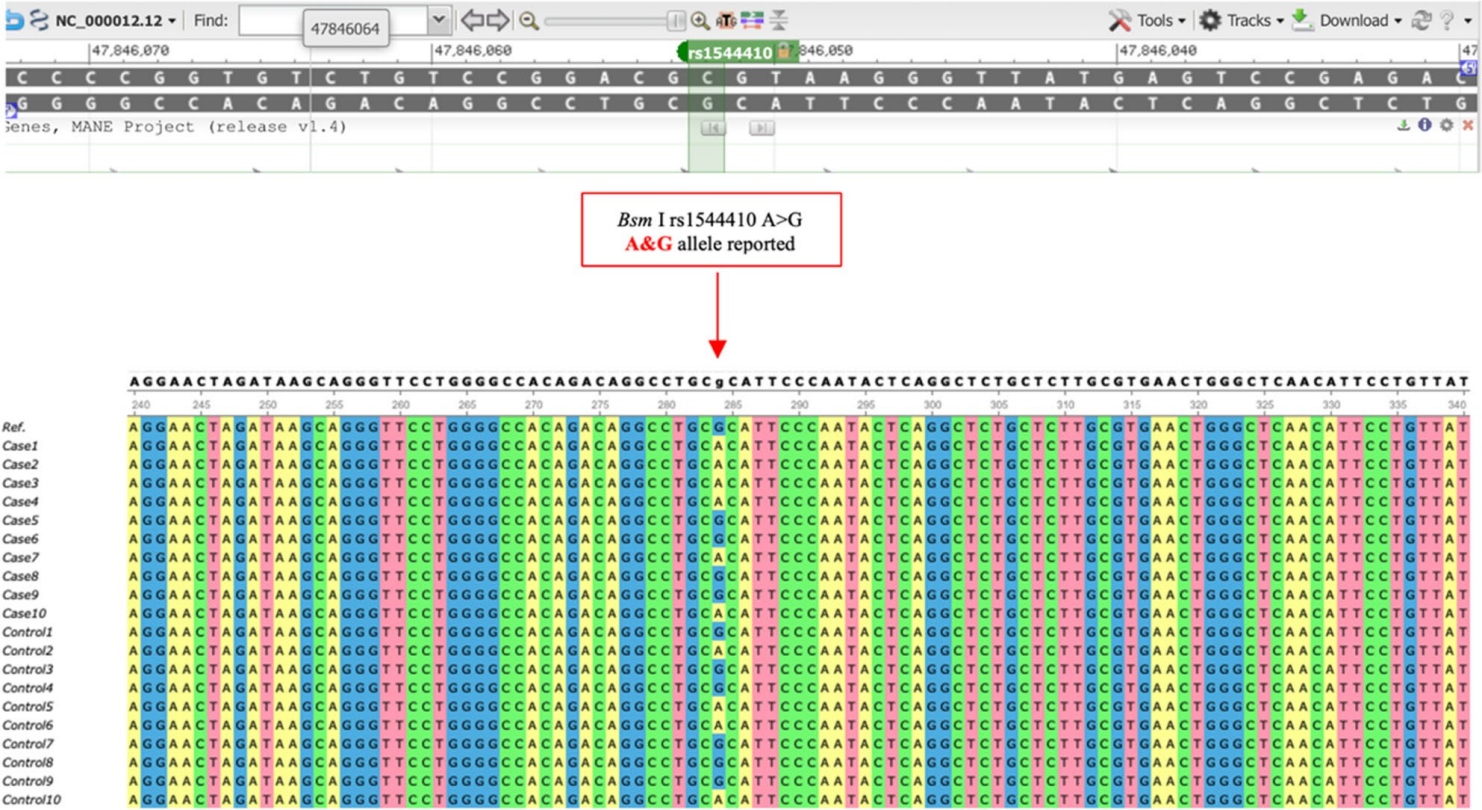
Sequence alignment of the VDR BsmI (rs1544410) polymorphism. Alignment displays A > G allelic substitution at chr12: 47,846,052 (GRCh37). The AA genotype was significantly more prevalent among OA patients compared to controls, suggesting a possible risk association

 In contrast, the BsmI polymorphism exhibited considerable variability. The genotype frequencies in the OA group were 48% AA, 31% AG, and 21% GG, whereas in the control group they were 29% AA, 42% AG, and 29% GG. Statistical analysis (Table 3) demonstrated a significant association between the AA genotype and osteoarthritis. Under the recessive model (comparing AA vs. AG + GG), the AA genotype was associated with an odds ratio (OR) of 2.26 (95% CI: 1.21–4.23, *p* = 0.006) Moreover, allele analysis revealed that the A allele was significantly more frequent in cases (63.5%) compared to controls (50%), corresponding to an OR of 1.74 (95% CI: 1.17–2.59, *p* = 0.007). (Fig. 2).

**Table 3:** Association of VDR *BsmI*(rs1544410)Polymorphism with Osteoarthritis

Genotypes	Frequencies%		*P* value	OR (95% CI)
	Case = 100	Control = 100		
AA	48%	29%	0.006*	2.26 (1.21–4.23)
AG	31%	42%		
GG	21%	29%		
Alleles				
A	127 (63.5%)	100 (50%)	0.007	1.74 (1.17–2.59)
G	73 (36.5%)	100 (50%)		

Odds ratio (OR) were calculated using univariate logistic regression under a recessive genetic model (*AA versus AG + GG), CI = confidence interval, NS = not significant, *P* value Chi-Square test

### *APa I* polymorphism (rs7975232)

Sequence alignment demonstrated that all OA cases and controls carried the G allele at the ApaI (rs7975232) locus. No allelic or genotypic variation was observed in the studied population, indicating that this locus was non-polymorphic. Consequently, no association analysis with osteoarthritis could be performed.

### *TaqI* polymorphism (rs731236)

**Fig. 3:**
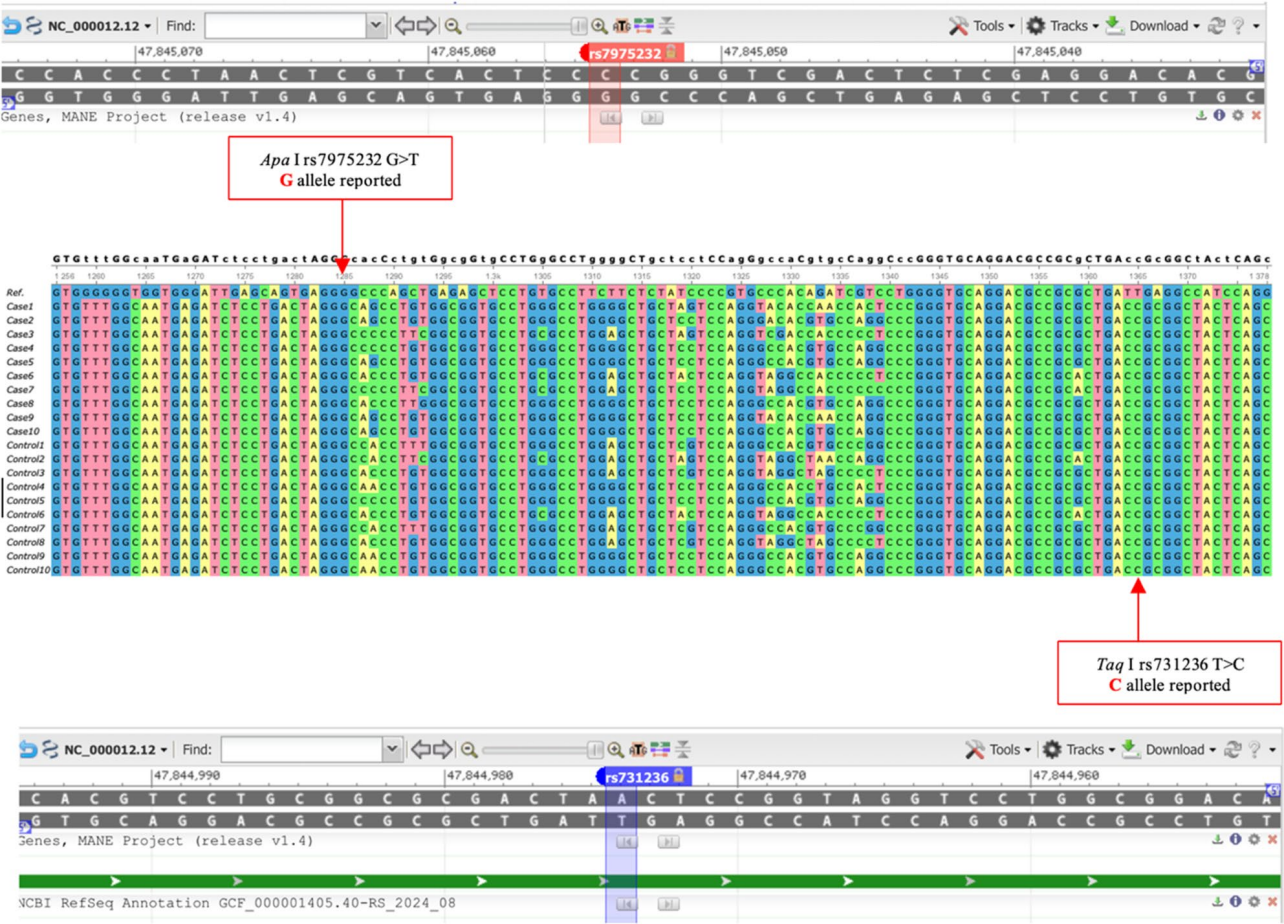
Sequence alignment of VDRApaI (rs7975232, G > T) and TaqI(rs731236, T > C) polymorphisms. Panels (A) and (B) show ApaI (G > T) and TaqI(T > C) substitutions, respectively.; ApaI & TaqI showed complete G-allele & C-allele fixation respectively. Reference sequences are shown on top; sample alignments below (GRCh37)

Sequence analysis showed that all study participants, including both OA cases and controls, carried the C allele at the TaqI (rs731236) locus instead of the reference T allele (Fig. 3). This uniform allele distribution indicates that the TaqI site was non-polymorphic in the studied population. Consequently, no association analysis with osteoarthritis could be performed for this locus.

## Discussion

This study investigated the association between common *VDR* gene polymorphisms and (OA) susceptibility in a Kurdish population. Four widely studied VDR variants (FokI, BsmI, ApaI, and TaqI ) were analyzed in a hospital-based case–control cohort comprising 100 OA patients and 100 healthy controls. Among the loci examined, only the BsmI polymorphism exhibited allelic and genotypic variability and showed a significant association with OA, whereas FokI, ApaI, and TaqI were non-polymorphic in this population. The BsmI polymorphism demonstrated a marked difference in genotype distribution between cases and controls, with the AA genotype significantly more prevalent among OA patients. Under a recessive genetic model (AA vs. AG + GG), this genotype was associated with a more than twofold increase in the odds of OA, and allele-level analysis further showed an increased frequency of the A allele among cases. These findings are consistent with previous reports linking BsmI variants to musculoskeletal disorders, including osteoporosis and osteoarthritis, suggesting a potential role for this locus in bone and cartilage metabolism [22–26]. Although the BsmI polymorphism is located within the 3′ untranslated region of the VDR gene, non-coding variants have been proposed to influence gene regulation rather than protein structure. In the present study, no structural modeling, mRNA stability prediction, or functional expression analyses were conducted; therefore, any proposed functional implications of the BsmI variant remain speculative. This cautious interpretation aligns with prior literature emphasizing that associations at non-coding loci require experimental validation to establish biological relevance [16]. In contrast, the FokI, ApaI, and TaqI loci were non-polymorphic in this cohort, with all participants carrying identical alleles at these sites. This finding differs from reference genomic databases and highlights the importance of population-specific genetic studies. Similar population-restricted genetic patterns have been documented in other complex diseases, reinforcing the concept that genetic associations may vary substantially across ethnic groups and disease subtypes [27]. The absence of variability at these loci rendered them uninformative for OA association analysis in the Kurdish population examined here. OA is increasingly recognized as a complex disorder driven by chronic low-grade inflammation, chondrocyte apoptosis, and progressive degradation of the extracellular matrix (ECM) [28]. Additionally, it has been described how inflammatory mediators promote apoptotic signaling in chondrocytes and accelerate ECM breakdown, thereby amplifying cartilage destruction and disease progression [28–30]. Pro-inflammatory cytokines such as IL-1β, TNF-α, and IL-6 stimulate matrix metalloproteinase activity and disrupt cartilage homeostasis, processes central to OA pathophysiology. Recent mechanistic studies have further expanded understanding of inflammatory regulation within the joint microenvironment. Moreover, synovial-derived extracellular vesicles can modulate inflammatory signaling and regulate chondrocyte apoptosis in inflammatory arthritis models, highlighting the importance of intercellular communication in cartilage degeneration [31, 32]. Although these findings are not directly related to VDR genetics, they provide a broader biological context for how immune-mediated mechanisms contribute to OA progression. Baseline comparisons between cases and controls in the present study revealed significant differences in sex distribution and serum vitamin D levels. The higher vitamin D levels observed in OA patients may appear counterintuitive given vitamin D’s proposed protective role in musculoskeletal health. Serum vitamin D concentrations may reflect post-diagnosis supplementation, clinical management, or increased health awareness rather than baseline vitamin D status prior to disease onset. Importantly, circulating vitamin D levels may not accurately reflect intracellular vitamin D activity or VDR-mediated signaling efficiency within joint tissues, which is more directly relevant to OA pathophysiology. Recent methodological advances support a nuanced interpretation of vitamin D-immune interactions. Mendelian randomization studies, such as the work on micronutrients and allergic disease risk [33], provide evidence for causal roles of micronutrients in immune-mediated conditions, offering a conceptual framework for investigating vitamin D-VDR signaling in chronic inflammatory disorders. In parallel, emerging insights into immunometabolic regulation highlight the interconnected roles of metabolic and immune pathways in disease susceptibility [34]. Genetic overlap across immune-mediated and inflammatory diseases has also been increasingly recognized. Description of autoimmune and inflammatory genetic backgrounds across diverse conditions, emphasizing overlapping regulatory pathways that influence disease risk [35]. Such observations provide a broader biological context for interpreting population-specific genetic associations, including those involving VDR polymorphisms, without implying direct mechanistic overlap or causality in OA. In addition to mechanistic advances, bibliometric analyses have documented a growing research focus on immune-related complications and genetic susceptibility in chronic diseases, including musculoskeletal disorders [36, 37]. These trends underscore increasing scientific interest in immune–genetic mechanisms underlying chronic degenerative conditions such as OA. The interventions between the gene-based and pathway-targeted aimed at modulating inflammatory signaling, cartilage metabolism, and joint homeostasis [38]. While such strategies are beyond the scope of the present genetic association study, they provide important future-oriented context for how population-specific genetic findings, such as VDR polymorphism profiles, may eventually inform personalized or mechanism-driven therapeutic research. Several limitations of this study should be acknowledged. The modest sample size limited statistical power and precluded stratified or multivariable analyses, particularly with respect to sex-specific effects. Functional validation of the BsmI polymorphism was not performed, and environmental factors influencing vitamin D status, such as sun exposure, dietary intake, and supplementation, were not systematically assessed. In addition, the cross-sectional case–control design does not permit causal inference. Larger, longitudinal studies incorporating functional assays and detailed environmental data are warranted to validate and extend these findings.

## Conclusion

This study highlights a potential association between the VDR BsmI polymorphism and osteoarthritis susceptibility in the Kurdish population. A significant association was observed for the BsmI AA genotype, whereas the FokI, ApaI, and TaqI loci were non-polymorphic in this cohort and therefore not informative for association analysis. Although the biological mechanisms underlying the BsmI association remain unclear, these findings contribute population-specific genetic data to the growing literature on VDR variation in osteoarthritis. Any functional or clinical implications remain speculative, and further studies incorporating functional validation, environmental exposure assessment, and larger cohorts are required to clarify the role of VDR polymorphisms in osteoarthritis pathogenesis.

## Data Availability

The datasets generated and/or analysed during the current study are available in the [NCBI] repository, [https://www.ncbi.nlm.nih.gov/SNP/snp_viewTable.cgi?handle=RAYA-YASHOOA].
